# Analysis of the early heterocyst Cys-proteome in the multicellular cyanobacterium *Nostoc punctiforme* reveals novel insights into the division of labor within diazotrophic filaments

**DOI:** 10.1186/1471-2164-15-1064

**Published:** 2014-12-04

**Authors:** Gustaf Sandh, Margareta Ramström, Karin Stensjö

**Affiliations:** Microbial Chemistry, Department of Chemistry - Ångström Laboratory, Science for Life Laboratory, Uppsala University, Box 523, SE-751 20 Uppsala, Sweden; Analytical Chemistry, Department of Chemistry - Biomedical Center, Science for Life Laboratory, Box 599, SE-751 24 Uppsala, Sweden

**Keywords:** Cyanobacteria, Nitrogen fixation, Cell differentiation, Heterocyst, Cys Proteome, ICAT, Thiols, Quantitative proteomics

## Abstract

**Background:**

In the filamentous cyanobacterium *Nostoc punctiforme* ATCC 29133, removal of combined nitrogen induces the differentiation of heterocysts, a cell-type specialized in N_2_ fixation. The differentiation involves genomic, structural and metabolic adaptations. In cyanobacteria, changes in the availability of carbon and nitrogen have also been linked to redox regulated posttranslational modifications of protein bound thiol groups. We have here employed a thiol targeting strategy to relatively quantify the putative redox proteome in heterocysts as compared to N_2_-fixing filaments, 24 hours after combined nitrogen depletion. The aim of the study was to expand the coverage of the cell-type specific proteome and metabolic landscape of heterocysts.

**Results:**

Here we report the first cell-type specific proteome of newly formed heterocysts, compared to N_2_-fixing filaments, using the cysteine-specific selective ICAT methodology. The data set defined a good quantitative accuracy of the ICAT reagent in complex protein samples. The relative abundance levels of 511 proteins were determined and 74% showed a cell-type specific differential abundance. The majority of the identified proteins have not previously been quantified at the cell-type specific level. We have in addition analyzed the cell-type specific differential abundance of a large section of proteins quantified in both newly formed and steady-state diazotrophic cultures in *N. punctiforme*. The results describe a wide distribution of members of the putative redox regulated Cys-proteome in the central metabolism of both vegetative cells and heterocysts of *N. punctiforme*.

**Conclusions:**

The data set broadens our understanding of heterocysts and describes novel proteins involved in heterocyst physiology, including signaling and regulatory proteins as well as a large number of proteins with unknown function. Significant differences in cell-type specific abundance levels were present in the cell-type specific proteomes of newly formed diazotrophic filaments as compared to steady-state cultures. Therefore we conclude that by using our approach we are able to analyze a synchronized fraction of newly formed heterocysts, which enabled a better detection of proteins involved in the heterocyst specific physiology.

**Electronic supplementary material:**

The online version of this article (doi:10.1186/1471-2164-15-1064) contains supplementary material, which is available to authorized users.

## Background

The filamentous cyanobacterium *Nostoc punctiforme* ATCC 29133 (from now on *N. punctiforme*) is a multicellular bacterium in which diverse cell-types can be formed by cell differentiation in response to various environmental stimuli [1, 2]. In order to survive times of combined nitrogen limitation *N. punctiforme* differentiate a new cell-type; the heterocyst, which is specialized in the fixation of atmospheric nitrogen (N_2_) into ammonium [3]. Heterocysts are formed at semi-regular intervals along filaments consisting of vegetative cells. The cell differentiation into a functional N_2_-fixing heterocyst takes about 24 hours and includes a significant reprogramming of the metabolism as well as morphological remodeling. The vegetative cells and the heterocysts are utterly interdependent, with the photosynthetic vegetative cells providing fixed carbon to the non-carbon fixing diazotrophic heterocysts, which receive fixed nitrogen in return [4–6]. The transformation into a diazotrophic culture therefore also includes a substantial metabolic reprogramming of the vegetative cells [7]. To protect the oxygen sensitive N_2_ fixation process heterocysts have a micro-oxic interior, formed through e.g. the lack of O_2_ evolving photosynthetic activity, increased respiration and the development of a thick envelope of polysaccharides and glycolipids [8]. Moreover, in the heterocysts both energy and reducing equivalents are efficiently directed to the nitrogenase enzyme complex, which catalyzes the N_2_ fixation reaction [9].

The gene expression patterns during the early stages of heterocyst differentiation have been analyzed in several heterocyst-forming cyanobacteria, e.g. [10–15]. Much work has focused on defining the differentially regulated transcriptome of filaments during heterocyst differentiation, i.e. up to 24 hours after combined nitrogen deprivation. In a recent study of *N. punctiforme*, it was shown that transcriptional changes detected in N_2_-fixing filaments, as compared to ammonium grown cells, are not completed within 24 hours after removal of combined nitrogen. Instead, it continues to change until a steady-state N_2_-fixing culture is established. In fact, over 800 genes showed a differential expression pattern in filaments from newly formed diazotrophic cultures, compared to steady-state cultures [12]. This indicates that the physiology of filaments containing newly formed functional heterocysts differ from filaments containing their steady-state counterparts. On the proteome level, heterocysts specific proteomes have been characterized, relatively quantified and compared to ammonium grown cultures from both *N. punctiforme*[7, 16, 17] as well as from its close relative *Anabaena* sp. PCC 7120 [18]. However, these studies are all on heterocysts isolated from steady-state diazotrophic cultures, and there is a lack of knowledge of the proteome landscape of a synchronized population of newly formed heterocysts.

Cell differentiation includes transcriptional regulation as well as proteome and metabolic regulation in which changes in the abundance of proteins/enzymes play a major role. However, the dynamics of the proteome comes not only with the vast number of proteins differentially expressed under certain conditions but also by the numerous activity regulating post-translational modifications that each of these proteins can undergo. Redox mediated modifications of protein bound thiols (Cys) are one important modification that regulates the activities of cellular proteins, both in *Escherichia coli*[19], yeast [20] and cyanobacteria [21]. In unicellular cyanobacteria changes in the availability of CO_2_ and combined nitrogen have e.g. shown to induce substantial changes in the redox state of the Cys-proteome [21, 22].

We hypothesized that newly formed functional heterocysts, present approximately 24 hours after combined nitrogen depletion, are still undergoing a metabolic adaptation. This goes both for its new internal physiology and its newly established mutualistic partnership with the surrounding vegetative cells. These early stage heterocysts are as a population more synchronized in age than the heterocysts present in a steady-state diazotrophic culture. Therefore, we also hypothesize that analyzing a more homogenous heterocyst population will enable a better resolution of the cell-type specific proteome of active heterocysts. These differences will include cell-type specific changes in the abundance of proteins and cell-type specific redox-mediated regulation, in which thiol-proteins are involved. We have as an initial step mapped the differential Cys-containing proteomes of two different cell samples of *N. punctiforme* (heterocysts as compared to their parental N_2_-fixing filaments) by quantitative proteomics using the cleavable ICAT reagent [23–26], in combination with high-resolution mass spectrometry (MS), i.e. Orbitrap MS. To our knowledge this represents the first application of ICAT proteomics in cyanobacteria and we have therefore characterized its quantitative accuracy in our complex protein samples. This study presents the first Cys-proteomes of two distinct cell-types in a bacterium. In addition, it constitutes the first report of the early cell-type specific heterocyst proteome (24 hours after combined nitrogen step-down) and complements existing data from *N. punctiforme*[7, 12, 27]. Notably, the study also report on proteins not previously quantified at the cell-type specific level, thereby expanding the coverage of the detected cell-type specific proteomes of *N. punctiforme* and the metabolic landscape of heterocysts in general.

## Methods

### Cell culturing and sampling

Two 400 mL axenic cultures of *Nostoc punctiforme* ATCC 29133-S (also known as UCD 153) [27, 28] were grown at 30°C under 50 μmol photons m^2^ s^-1^ with aeration in BG11 lacking nitrate (BG11_0_) [7], supplemented with 10 mM NH_4_Cl and 20 mM HEPES pH 7.4. After 7 days the cells were washed twice in BG11_0_ before being re-suspended into 1.6 L BG11_0_ in 2 L E-flasks with both stirring and aeration at an initial chlorophyll concentration of 1 μg Chl *a* mL^-1^. After 24 hours, heterocyst formation was confirmed by staining cells with an equal volume of 0.5% (w/v) alcian blue (dissolved in 50% ethanol) for 10 min. The cells were inspected using an Axiostar plus light microscope (Zeiss). After detection of heterocysts in the cultures 50 mL from each culture was harvested by centrifugation (5 min, 3500 g) and the pellet was resuspended in 500 μL denaturing alkylation buffer (DAB: 6 M Urea, 0.5% SDS, 10 mM EDTA in 200 mM Tris–HCl pH 8.5) [19], frozen in liquid N_2_ and stored as the parental N_2_-fixing filament fractions at -80°C until further processing. The remaining N_2_-fixing cultures were then harvested as above. Heterocysts were isolated as in Ow *et al.*[7], using lysozyme and sonication to break the weaker cell walls of the vegetative cells. The final heterocyst fractions were inspected by light microscopy, resuspended in 1 mL DAB buffer, frozen in liquid N_2_ and stored as the heterocyst fractions at -80°C until further processing.

### Protein extraction, isotope coded affinity tag (ICAT) labeling and trypsination

The samples were thawed and acid washed glass beads were added prior to bead beating in a Precellys 24 (Bertin Technologies), 3 times (5500 g, 30 sec) with 1 min on ice in-between. After pelleting any remaining cell debris (4 min, 20000 g, 4°C) the supernatant was transferred to a new eppendorf tube and a small aliquot was removed for determining the protein concentration. The samples were then frozen in liquid N_2_ and stored at -80°C until further processing. The protein concentrations were determined using the Bio-Rad RC DC kit with a bovine serum albumin standard as reference. Labeling of the protein samples were performed according to a modified protocol from the one described in Leichert *et al.*[19]. In short, 100 μg of each sample were diluted to 80 μL in DAB buffer and reduced by 2 μL TCEP (tris (2-carboxyethyl) phosphine) before one vial of cleavable ICAT reagent (light or heavy, dissolved in 20 μL acetonitrile (ACN)) was added to each sample. The labeling reaction was performed in darkness at 37°C, 250 rpm for 2 h. In the initial test of the accurate quantitation, light and heavy cleavable ICAT labeled total proteins from N_2_-fixing filaments were used and mixed 1:1 (v/v), 5:1 (v/v) and 1:5 (v/v). For the cell-type specific samples the entire volume of the total proteins from N_2_-fixing filaments labeled with the heavy cleavable ICAT reagent were mixed with the entire volumes of the heterocyst fractions labeled with the light cleavable ICAT reagent. After pooling the proteins were precipitated by adding five volumes of ice-cold acetone and stored at -20°C for 4 h. After pelleting the proteins (10 min, 13000 g, 4°C), trypsination was performed by resuspending the proteins in 200 μL ICAT denaturing buffer (in 20% ACN; AB SCIEX) and adding one aliquot trypsin (resuspended in 200 μL H_2_O; AB SCIEX). The samples were incubated at 37°C for 14 h. The samples were then mixed by vortexing before the entire volume of the accurate quantitation samples and 10% (v/v; 40 μL) of the cell-type specific samples were acidified by adding the cleavable ICAT kit cation exchange loading buffer (AB SCIEX) until pH 3 and stored at -80°C.

### Peptide fractionation and strong cation exchange (SCX) liquid chromatography (LC)

The remaining fraction (90%) of the cell-type specific Cys-protein samples were subjected to peptide fractionation by SCX using a PolySULFOETHYL A Pre-Packed Column (PolyLC) with a 5 *μ*m particle size, a column dimension of 200 mm × 2.1 mm i.d. and 200 Å pore size connected to a ACQUITY UPLC System (Waters). SCX was performed as in Ow *et al.*[7] but with a few modifications. We used a multi-step salt gradient using buffer A (10 mM KH_2_PO_4_, 25% ACN, pH 2.85) and buffer B (500 mM KCl, 10 mM KH_2_PO_4_, 25% ACN, pH 2.85). Samples were loaded in 100% buffer A. The separation started with a linear gradient from 0 to 8.4% B for 14 min followed by an incremental gradient from 8.4% to 12.4% B for 6 min. Next followed a linear gradient 12.4% to 20% B for 15 min and a linear gradient 20% to 40% B 10 min before a final linear gradient from 40% to 100% B for 10 min. Samples were collected in 1 mL fractions. The column was washed with buffer C (1 M KCl, 10 mM KH_2_PO_4_, 25% ACN, pH 2.85) for 20 min and reconditioned in buffer A in-between each run. In total 320 μL was loaded per sample and the liquid flow rate was 0.2 mL min^-1^. The 19 collected samples were pooled into 10 samples based on the signal intensity of the eluted fractions at 214 nm. The sample pH was neutralized by addition of the cleavable ICAT reagent kit affinity loading buffer (AB SCIEX) and were stored at -20°C until further processing. For the samples not subjected to peptide fractionation, SCX was performed according to the supplier’s instructions for the Cleavable ICAT Reagent Kit (AB SCIEX).

### Purification of ICAT-labeled peptides

The labeled peptides were then further purified using Avidin affinity chromatography, followed by cleavage of the Biotin linker, according to the manufacturer’s instructions (Cleavable ICAT Reagent Kit; AB SCIEX). The eluted peptides were dried using a Concentrator 5301 vacuum centrifuge (Eppendorf) and stored at -20°C.

### Nano-LC and mass spectrometry

The peptides prepared for the initial quantitation accuracy test were dissolved in 100 μL 0.1% formic acid (FA), while the peptides obtained after fractionation were dissolved in 15 μL 0.1% FA. Five μL of each sample was injected on an EASY-nanoLC-system (Thermo Scientific). The enzymatic peptides were separated in reversed phase on a 10 cm long C18-A2 column, ID 75 μm (Thermo Scientific) using mobile phase A (0.1% FA) and B (0.1% FA, 99.9% ACN). The gradient used was 4-50% B in 60 min, followed by a steep gradient to 80% B. The peptides were electrosprayed on-line to a LTQ-Orbitrap Velos Pro ETD Mass spectrometer (Thermo Scientific). Tandem mass spectrometry (MS/MS) was performed applying collision-induced dissociation.

### ICAT data analysis

Proteome Discoverer 1.3 (Thermo Scientific) was applied for data evaluation. The peptides were matched towards proteins in the *Nostoc punctiforme* ATCC 29133 protein database. The annotations used in the searches were downloaded from PATRIC (http://patricbrc.org) [29]. The Sequest search engine was applied for peptide mapping. A tandem search was applied using the Mascot search engine and proteins quantified, i.e. peptides only detected, using the Mascot search engine were counted but labeled Mascot in Additional file 1. Using either method, the precursor mass tolerance was set to 10 ppm and the fragment mass tolerance was set to 0.8 ppm. The light and heavy labels, ICAT-C (+227.127 Da) and ICAT-C:13C(9) (+236.157 Da), were set as dynamic modifications for all Cys residues in the samples. Methionine oxidation was also included as a dynamic modification. Peptides scoring at medium confidence or higher, corresponding to a false discover rate ≤0.05, were considered as true matches. Peptide ratios of light/heavy ICAT were automatically calculated from labeled peptide pairs of the same charge state by dividing the corresponding precursor ion areas.

### Protein metadata analysis

The available metadata of all proteins in the PATRIC annotation was downloaded from http://patricbrc.org and the E.C. numbers were used to reconstruct metabolic maps using KEGG mapper (http://www.genome.jp/kegg/tool/map_pathway2.html). In addition, the subcellular localization of all proteins were predicted using the PSORTb v3.0.2 software (http://www.psort.org) and Mw and pI data for the entire theoretical proteome were generated by the pI/Mw predication tool [30, 31]. Manually curated functional annotation categories [27] were also included to better group proteins into cyanobacterial and heterocyst related metabolic processes.

## Results and discussion

### ICAT method evaluation

**Figure 1:**
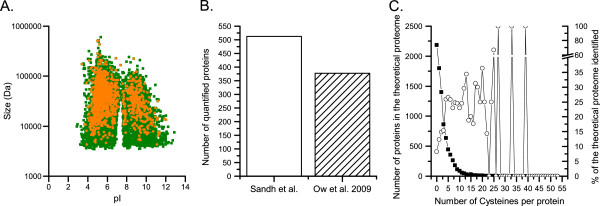
**Distribution and quantity of the detected proteins. (A)** The distribution of the theoretical proteome and identified proteins based on their respective size and charge. **(B)** The number of relatively quantified proteins in cell-type specific (Heterocysts/N_2_-fixing filaments) quantitative proteomic analyses of *N. punctiforme*. Only proteins detected in all biological replicates in the present study (Sandh *et al.*) and from Ow *et al.*[7] are included. **(C)** The distribution of proteins with different abundance of cysteines in the theoretical and the identified Cys-proteome of *N. punctiforme*. The number of cysteines per protein in the theoretical proteome are indicated by black squares. The percentage of identified proteins within each group of proteins (containing a defined number of cysteines) are indicated by open circles. Note that the sporadic higher coverage of protein groups consisting of proteins with over 25 cysteines per protein is due to very few proteins per group.

We here evaluated the performance of the selected ICAT strategy on the total Cys-proteomes of both whole N_2_-fixing filaments and isolated heterocysts, prior to and after off-line peptide fractionation by SCX. In total, using peptide fractionation and a sensitive LTQ-Orbitrap mass spectrometer, 1344 proteins were identified (data not shown). Of these, 511 proteins were labeled by both the light and heavy isoform of ICAT, in both biological replicates. This allowed us to perform a relative quantification of their abundance in isolated heterocysts as compared to whole N_2_-fixing filaments (Additional file 1). The strategy to compare the abundance of proteins in isolated heterocysts compared to in whole N_2_-fixing filaments has previously been applied in a few studies [7, 18]. It provides an indication of the division of labor between heterocysts and the vegetative cells in a diazotrophic culture, at a given time point. However, it doesn’t give information on changes in the absolute protein concentrations between different growth conditions, or time points. The use of peptide fractionation before the LC-MS/MS analysis enhanced the number of both identified and relatively quantified proteins by approximately 150%. The identified proteins span the entire scale of size (M_w_) and charge (pI), compared to the theoretical proteome. As previously seen in quantitative proteomic analyses see e.g. [32] a lower percentage of hydrophobic proteins and low molecular weight proteins were detected, compared to larger and more hydrophilic proteins (Figure 1A). This is likely due to shortcomings in the available sample preparation procedures. The use of the PATRIC gene annotation lead to an increased detection of proteins in our initial experiments, compared to the standard RefSeq annotation (data not shown), and was therefore used throughout this study. The differences between the two annotations are in gene calling, the location of stop and start sites and the removal of certain types of regulatory sequences. The theoretical PATRIC proteome is substantially larger than the standard theoretical RefSeq proteome and it should be pointed out that most of the low molecular weight proteins annotated in the theoretical *N. punctiforme* PATRIC proteome have to date not been detected in any proteomic analyses.

Cysteines constitute less than 1% of the amino acids in the theoretical proteome of *N. punctiforme*[33]. One drawback of the Cys-selective labeling strategy, when mapping changes in the total proteome, is therefore that the Cys-containing proteome only covers ~75% of the theoretical proteome. In spite of this aspect we were, in comparison to previous isobaric Tags for Relative and Absolute Quantitation (iTRAQ) based studies of *N. punctiforme*[7], able to in total surpass the amount of relatively quantified proteins (Figure 1B). 65% of the relatively quantified proteins in the present study (Additional file 1) have not previously been quantified at the cell-type specific level in *N. punctiforme*. However, close to 50% of these proteins have previously been identified in *N. punctiforme* cells [17]. This suggests that part of the detected differences is based on changes in protein composition and complexity while others could be due to differences in the labeling methodologies used. The proteins quantified in the present study were enriched for peptides containing cysteines and thereby the sample complexity is shifted from the total abundance of all peptides to the selective abundance of the Cys-enriched peptide pool. In general proteins containing multiple (≥4) Cys residues per protein were identified at a higher percentage relative to the theoretical proteome, as compared to proteins with one or only a few cysteines (Figure 1C). Another important factor, which would have an impact on the differences found between the iTRAQ [7] and ICAT studies, is that they describe differences in cell-type specific proteomes of cultures at different time points following the removal of combined nitrogen. The complementary nature of the different labeling methods therefore needs further investigation. In the present study, we report the relative abundance of 189 proteins, not previously quantified in *N. punctiforme*, neither on the proteomic nor on the transcriptomic level, in cultures grown with and without combined nitrogen (Additional file 2). Thus, the results from this investigation have increased the general insight into the proteome and physiology of heterocysts.

ICAT has earlier been shown to provide a very good quantitative accuracy using less complex protein samples [24]. To test the quantitative performance on our complex protein samples*,* we analyzed peptides, labeled with either heavy or light cleavable ICAT, mixed in different defined ratios. The analyzed samples consisted of the total Cys-enriched peptide pool from N_2_-fixing filaments. The detected relative ratios at the protein level showed high quantitative accuracy, calculated from hundreds of proteins from each sample (Table 1). The 1:1 and 1:5 dilutions showed slightly lower median values (0.90 and 0.16, respectively) compared to the theoretical values (1.0 and 0.20, respectively). In contrast the 5:1 dilution showed a slightly higher median (5.3) compared to its theoretical value (5.0) and a higher variance (28%). The results show that ICAT within this quantitative range provides a good quantitative accuracy, with a low compression bias of quantified ratios. The latter is a problem previously identified with iTRAQ based quantitative proteomics [34, 35]. However, the increased variance at higher ratios emphasizes caution when trying to assign proteins as being exclusive to the heterocysts, using this technique alone. In our subsequent analysis, we used a 1.6-fold threshold to signify proteins more abundant in heterocysts compared to whole N_2_-fixing filaments, or vice versa. The selection of 1.6-fold as cut-off is supported both by our own pilot study and from literature [32]. The 5th percentile value for the 1:1 dilution given in Table 1 corresponds to a P-value of 0.05, i.e., 5% false positive. The value, 0.657, corresponds to a down-regulation of 1.52 and therefore 1.6 was chosen as cut-off. All individual ratios from each of the two biological replicas, were for all proteins located above or below the 1.6-fold cut-off, clearly separated from 1, indicating a relatively low technical and biological variation (data not shown).

**Table 1:** **Quantitative accuracy of the cleavable ICAT reagent in complex samples**

Mixed ratio (light: heavy)	1:5	1:1	5:1
Expected	0.2	1.0	5.0
Average	0.168	0.882	5.41
Median	0.159	0.895	5.31
Stdev	0.088	0.142	1.24
95th percentile	0.227	1.093	7.73
5th percentile	0.103	0.657	3.63
Variance	0.008	0.020	1.53
Variance %	4.83	2.26	28.8
Number of proteins analyzed	146	213	150

Relative abundance ratios of total proteins isolated from N_2_-fixing filaments of *N. punctiforme* labeled with the light- or heavy-ICAT reagent and mixed in 1:1, 1:5 and 5:1 ratios.

In addition to the more confident quantitative accuracy when using the cleavable ICAT reagent, a broader range of quantified ratios was also detected, as compared to previous studies [7] (Additional file 3). The heterocyst specific staining revealed an approximate heterocyst frequency of 7.5% within the newly formed N_2_-fixing filaments. The presented ICAT data correlates very well with the upper theoretical biological limit of the relative ratio (13.3-fold) of proteins exclusively expressed in heterocysts (inherit with the detected heterocyst frequency). The lowered sample complexity of our Cys-peptidome could be a factor of importance for the improved performance. Decreased sample complexity due to sample fractionation has previously been shown to alleviate the compression of ratios, which has been identified as a problem in iTRAQ based quantitation [34–36]. The biological differences between steady-state [7] and newly induced diazotrophic cultures, where the heterocysts are in a similar physiological phase, could also play a role.

### The cell-type specific Cys-proteome of newly formed heterocysts

Predictions of cellular location of the identified Cys-proteins showed that a low proportion of the relatively quantified proteins consisted of proteins from the cytoplasmic membranes, and proteins of unknown localization, compared to the theoretical proteome. In contrast, a higher percentage of cytoplasmic proteins were quantified while the other fractions matched the percentage in the theoretical proteome (Additional file 1). Overall, 74% of the quantified proteins showed a cell-type specific differential abundance (Table 2). Using previously published manually curated functional annotations of the relatively quantified proteins; it becomes apparent that a large part of all proteins detected at higher abundance in heterocysts belong to the adaptive metabolism [27]. In contrast, the majority of the proteins found at higher levels in the vegetative cells, which dominate the N_2_-fixing filaments, belong to the core metabolism or to the unassigned metabolism [27]. These results are not surprising as all proteins involved in N_2_ fixation, nitrogen assimilation as well as in heterocyst development are annotated as part of the adaptive metabolism. What is more striking is the strong general lower abundance in newly formed heterocysts of proteins involved in major core biosynthetic pathways, e.g. amino acid, cofactor, aminoacyl-tRNA and nucleotide biosynthesis. These results are validated by earlier biochemical, as well as proteomic studies in which heterocyst-enriched proteins have been identified see e.g. [7, 8, 10–14, 18]. In addition, the results are likewise corroborated by the lower relative abundance in the isolated heterocysts of proteins involved in pathways that have previously been shown to be absent or strongly down-regulated in heterocysts. Some examples are proteins involved in CO_2_ fixation, cell division and the proteins of the phycobilisome (Table 2; Additional file 1). A more detailed analysis of specific functional categories is given below. The aim of the following sections is to further explore the metabolic pathways that show cell-type specific differences, with focus on proteins not earlier discussed in a quantitative proteomic context (Table 2; Additional file 1). These detailed descriptions are justified based on the large degree of novel cell-type specific quantitative proteomic data presented in the current study. Nevertheless, many of the cellular processes are clearly validated by previously published results see e.g. [7, 8, 10–14, 18]. All values given are median ratios based on the relative quantification of proteins identified as differentially abundant in isolated heterocysts as compared to whole N_2_-fixing filaments.

**Table 2:** **Comparative functional characterization of quantified proteins**

		24 h (Present study)							Steady-state [7]						
***Function***		Total	>Het	% total	Equal	% total	>Fil	% total	Total	>Het	% total	Equal	% total	>Fil	% total
Adaptive metabolism	A	52	30	58	10	19	12	23	19	8	42	7	37	4	21
Core metabolism	C	292	43	15	76	26	173	59	236	60	25	87	37	89	38
Transport metabolism	T	16	5	31	9	56	2	13	13	7	54	5	38	1	8
Selfish metabolism	S	1	0	0	0	0	1	100	0	0	0	0	0	0	0
Unassigned metabolism	U	149	28	19	39	26	82	55	108	36	33	38	35	34	31
Not annotated	NA	1	0	0	0	0	1	100	1	1	100	0	0	0	0
Total		511	106	21	134	26	271	53	377	112	30	137	36	128	34
***Sub-function***															
Adaptive metabolism															
Carbon	A	3	0	0	1	33	2	67	1	0	0	0	0	1	100
Nitrogen	A	14	13	93	0	0	1	7	5	3	60	2	40	0	0
Development	A	16	15	94	1	6	0	0	3	2	67	1	33	0	0
Signal transduction	A	7	1	14	2	29	4	57	4	2	50	1	25	1	25
Stress	A	2	1	50	1	50	0	0	2	0	0	0	0	2	100
Taxis	A	1	0	0	0	0	1	100	0	0	0	0	0	0	0
Transcriptional regulation	A	6	0	0	5	83	1	17	2	1	50	1	50	0	0
Energy, monomer, polymer	A	2	0	0	0	0	2	100	2	0	0	2	100	0	0
Secondary	A	1	0	0	0	0	1	100	0	0	0	0	0	0	0
Core metabolism															
Precursor	C	53	3	6	16	30	34	64	57	11	19	13	23	33	58
Cofactor	C	21	1	5	3	14	17	81	6	1	17	1	17	4	67
Energy	C	20	9	45	6	30	5	25	32	21	66	9	28	2	6
Monomers	C	54	5	9	4	7	45	83	23	1	4	10	43	12	52
Polymers	C	85	14	16	33	39	38	45	77	12	16	42	55	23	30
Cell envelope	C	29	11	38	5	17	13	45	20	6	30	6	30	8	40
Cell division	C	5	0	0	2	40	3	60	3	1	33	1	33	1	33
Reactive oxygen species/metabolic protection	C	15	0	0	4	27	11	73	15	7	47	3	20	5	33
Storage	C	10	0	0	3	30	7	70	3	0	0	2	67	1	33
Selfish metabolism															
Phage	S	1	0	0	0	0	1	100	0	0	0	0	0	0	0
Transport metabolism															
ABC systems	T	11	3	27	6	55	2	18	6	3	50	3	50	0	0
Ion ATPase	T	1	1	100	0	0	0	0	3	2	67	0	0	1	33
Export	T	3	0	0	3	100	0	0	4	2	50	2	50	0	0
Porter	T	1	1	100	0	0	0	0	0	0	0	0	0	0	0
Unassigned metabolism															
Conserved hypothetical	U	81	16	20	21	26	44	54	57	21	37	18	32	18	32
Hypothetical	U	11	1	9	1	9	9	82	5	1	20	2	40	2	40
Unassigned	U	57	11	19	17	30	29	51	46	14	30	18	39	14	30
Not annotated	NA	1	0	0	0	0	1	100	1	1	100	0	0	0	0

The relative abundance levels of proteins in isolated heterocyst vs. their parental N_2_-fixing filaments 24h (present study) and 6 days [7] after combined nitrogen step-down. The quantified proteins were grouped based on their functional annotation [27] and their relative differential abundance levels (using the Log_2_ +/-0.678 thresholds of 1.6-fold). >Het (higher in heterocysts); >Fil (higher in filaments, i.e. vegetative cells) and Equal (even distribution) indicate the individual proteins cell-type specificity using the thresholds defined in the two studies.

#### Cell-type specific regulation of proteins involved in the adaptive metabolism: signal transduction, heterocysts development and nitrogen metabolism

A higher abundance of NtcA (Npun_F5511, 8.49-fold) was detected in the newly formed heterocyst proteome, which is in accordance with earlier shown transcriptional regulation of NtcA during nitrogen limitation [12]. NtcA is a transcription factor that regulates a large number of genes and under these conditions starts a signaling cascade, which involves both regulatory proteins as well as proteins involved in the general response to combined nitrogen limitation and those involved in heterocyst differentiation and heterocyst specific metabolism [37]. The result is not surprising considering that in steady-state N_2_-fixing cultures, more than 50% of the proteins found in higher abundance in heterocysts contain a putative NtcA binding site upstream of their corresponding gene [7].

**Figure 2:**
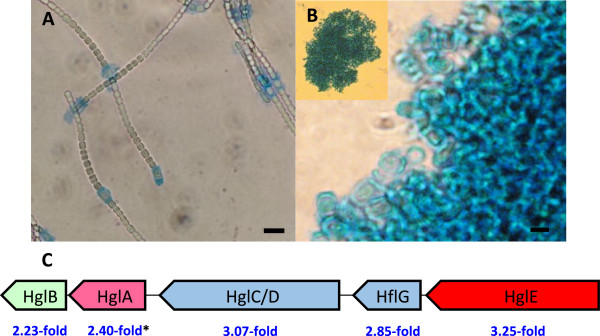
**Visualization of the heterocyst envelope and the cell-type specific relative abundance levels of proteins encoded by the** ***hgl*** **gene cluster.** Staining of **(A)** N_2_-fixing filaments and **(B)** the isolated heterocyst fraction from the same diazotrophic culture with (5%) alcian blue. A high degree of heterocyst purity was evident in the isolated fraction. **(A)** Size bar =10 μm, **(B)** Size bar =5 μm. **(C)** All proteins encoded within the *hgl* gene cluster (Additional file 1) showed higher relative protein abundance in heterocysts. The cell-type specific protein ratios are given as Log_2_ values (heterocysts/N_2_-fixing filaments) from samples taken 24 hours after the removal of combined nitrogen. *Indicates that the protein was detected in only one of the two biological replicas.

Heterocyst differentiation involves the formation of a thick envelope that surrounds the outer membrane and restricts gas diffusion that would otherwise interfere with N_2_ fixation, see e.g. [38–40] (Figure 2). The envelope consists of an inner layer of glycolipids surrounded by a layer of compacted followed by a layer of uncompacted polysaccharides. Two HetR regulated gene clusters, which encode for proteins involved in the synthesis of heterocyst envelope-specific glycolipids (the *hgl*-gene cluster) and polysaccharides (the *hep*-gene cluster), are present in the genomes of *N. punctiforme* and other heterocyst-forming cyanobacteria. All the proteins encoded in the *hgl*-gene cluster (HglE-HglB; Npun_R0043 - Npun_R0039) as well as several additional proteins involved in the biosynthesis of heterocyst specific glycolipids (DevA, DevH and HglT) [41–43] were found at higher abundance (4.70-22.32-fold) in the newly formed heterocysts (Figure 2, Additional file 1). In addition, a large number of proteins encoded by genes inside and surrounding the not so well defined *hep*-gene cluster [44], involved in the synthesis of the polysaccharide layer of the heterocyst envelope, were also more abundant in the newly formed heterocysts (2.18-10.77-fold; Additional file 1). These included the ABC transporter subunit HepA (Npun_R1068), a number of glycosyl transferases (Npun_R1071, Npun_R1072, Npun_R1073), as well as Npun_R1075, Npun_R1077 and Npun_R1079. In accordance with our results additional proteins encoded by genes outside of the *hep* gene cluster have also been shown to be required for the formation of heterocyst polysaccharides [45], e.g. the genes encoding the orthologs of Npun_F4337 and Npun_F2954, which were also found at higher abundance (4.90-10.10-fold) in the newly formed heterocysts. Compared to previous cell-type specific proteomic studies of steady-state cultures of both *N. punctiforme* and *Anabaena* sp. PCC 7120, this represents a much higher coverage of proteins involved in the heterocyst envelope [7, 18]. This could be indicative of the early heterocyst proteome, as earlier transcriptomic studies have shown a decreased expression of genes involved in heterocyst envelope development in isolated heterocysts from steady state cultures [15].

Other cell-type specific differences in the abundance of proteins involved in the envelope, cell wall and/or membrane biogenesis were also evident, with a large number of proteins with both higher and lower abundance in heterocysts (Additional file 1). The data indicates that several novel membrane proteins are involved in the development and the physiology of heterocysts. We also detected a higher abundance, in heterocysts, of proteins involved in the synthesis of other types of membrane constituents e.g. phospholipids (2.06-2.14-fold) and fatty acids (1.75-4.03-fold; Additional file 1). Genes involved in fatty acid and phospholipid synthesis have previously been seen to be up-regulated at the transcript level in N_2_-fixing filaments of *N. punctiforme*[12, 27]. The formed fatty acids are likely used in the synthesis of the new membrane constituents described above. The signal peptide peptidase SppA (Npun_F4271) also showed a higher abundance (2.77-fold) in the heterocysts, which could imply an increased membrane transport associated with the synthesis of the heterocyst envelope.

We identified an increased abundance of several proteins involved in N_2_ fixation in the newly formed heterocysts (Table 2; Additional file 1). In addition to all the subunits of the nitrogenase enzyme complex (NifHDK; 8.45-11.39-fold) we were also able to detect a higher abundance in the heterocysts (6.26-14.72-fold) of a large number of accessory proteins involved in the assembly and function of the nitrogenase enzyme complex (NifO, NifS, NifB, NifV, NifU, HesA, HesB and Npun_R0389). This indicates the presence of functional heterocysts 24 hours after combined nitrogen depletion, in the analyzed cultures. Again, this represents a higher coverage of proteins with a clear heterocyst specific function, as compared to previous cell-type specific proteomic studies of both *N. punctiforme* and *Anabaena* sp. PCC 7120 [7, 18].

Among all the metabolic enzymes in the presented dataset, proteins involved in amino acid metabolism formed the largest subsection (Additional file 1). More than 30 of the proteins involved in amino acid metabolism showed a lower abundance in heterocysts as compared to N_2_-fixing filaments. The only enzyme found at higher abundance in the heterocysts (1.83-fold) was alanine dehydrogenase (Npun_R3990). Recently, the transcript of alanine dehydrogenase has been shown to be up-regulated in the heterocysts of *Anabaena* sp. PCC 7120 [46] and in N_2_-fixing filaments of *N. punctiforme*[12]. This result support the earlier suggestion that alanine is transported from the surrounding vegetative cells into the heterocysts [47], where alanine dehydrogenase would convert the transported alanine into pyruvate and thus provide the heterocyst metabolism with reducing equivalents. Pernil *et al.*[46] showed that the pyruvate produced from alanine metabolism in the heterocysts is, in part, converted into glutamine, glutamate and aspartate, although the amidotransferase responsible for the formation of glutamate has not been described. In the present study two glutamine amidotransferases were identified, PyrG (Npun_F6033) and Npun_R3558. Both were found at higher abundance (1.59-2.35-fold) in heterocysts and with a clear differential abundance compared to their corresponding biosynthetic pathways (nucleotide and porphyrin biosynthesis, respectively), which showed a much lower abundance in the heterocysts as compared to their parental N_2_-fixing filament. Thus, these glutamine amidotransferases are possibly involved in the nitrogen metabolism of heterocysts.

#### Cell-type specific regulation of proteins involved in the core metabolism: Energy conversion, electron transport and carbon metabolism

The overall lower abundance of proteins involved in the core metabolism in the heterocysts, as compared to in their parental filaments, is not representative for proteins involved in respiration, ATP synthesis or photosynthesis (with the exception of pigment biosynthesis) (Table 2; Additional file 1). We detected higher abundance in heterocysts of several proteins involved in respiration (NDH-1; 1.82-18.79-fold), ATP synthesis (1.65-fold) and photosystem I (PSI) (1.58-1.93-fold). These results agree with earlier studies of different heterocyst-forming cyanobacteria [7, 12, 48]. Many of the subunits of cytochrome c oxidase and ATP synthase lack or contain very few Cys-residues, and we therefore conclude that the low coverage of proteins involved in theses pathways, compared to previous cell-type specific proteomic studies [7, 18], are likely due to methodological differences.

Proteins related to the light harvesting phycobilisomes were in general detected at lower abundance (0.53-0.11-fold) in the heterocysts. In addition, a large number of proteins involved in chlorophyll biosynthesis were also found at lower abundance (0.65-0.15-fold) (Additional file 1). Both porphyrin and phycobilisome biosynthesis have previously been shown to be down-regulated at the transcript level in N_2_-fixing filaments [12]. In addition, our results positively correlate with the well-documented decrease in pigment and phycobiliprotein content, which take place during heterocyst differentiation [49, 50].

It is well established that there are major changes occurring in the photosynthesis of vegetative cells and heterocysts during the transition from growth on ammonium to N_2_-fixing growth [49]. As mentioned above, our results correlate well with what has previously been reported, on the protein level. However, our data also includes some novel findings. Among others we detected a cell-type specific differential abundance of two response regulators, RpaA (Npun_F3659) and RpaB (Npun_F6321). In comparison to the parental N_2_-fixing filaments, RpaA was more abundant in the newly formed heterocysts (2.28-fold), while RpaB (Npun_F6321) showed a lower abundance in the isolated heterocysts (0.34-fold). The differential regulation of RpaA and RpaB has previously been shown to alter the direction of the flow of energy from the phycobilisomes, to either PSI or PSII, in *Synechocystis* sp. PCC 6803 [51]. The higher abundance of RpaA, in combination with the lower level of RpaB, found in the early heterocysts, therefore suggests a redirected energy transfer to PSI. In addition, higher levels of *rpaA* transcript have also been detected following nitrogen deprivation in filaments of *N. punctiforme*[11]. RpaA/RpaB regulation has also been linked to the circadian clock and the maintenance of basic transcription patterns in cyanobacteria [52–54]. Altogether, the potential functions of RpaA/RpaB are unclear, and therefore further investigations are needed to resolve their specific roles in the physiology of heterocysts.

The genome of *N. punctiforme* encodes a large number of different ferredoxins [55, 56]. Two previously reported heterocyst specific ferredoxins, FdxH (Npun_R0380, a 2Fe-2S ferredoxin) [57] and FdxB (Npun_F0335, a 4Fe-4S ferredoxin) [58] were both found at higher abundance (3.78-3.82-fold) in the heterocysts. In contrast, several other ferredoxins showed an opposite response, with six 2Fe-2S ferredoxins and a plastocyanin all found at lower abundance (0.35-0.13-fold) in the heterocysts as compared to their parental N_2_-fixing filaments (Additional file 1). A lower abundance of plastocyanin as well as differential abundance of ferredoxins has also been detected in heterocysts from steady-state N_2_-fixing cultures [7]. This indicates a clear difference in proteins involved in the basal electron transfer mechanisms in the two different cell-types of a diazotrophic culture of *N. punctiforme*, consequently altering the affinities associated with electron uptake and release within the vegetative cells and heterocysts.

Four enzymes involved in the oxidative pentose phosphate (OPP) pathway (Npun_F4023, Npun_R6307, Npun_F4025 and Npun_F4026) were found at higher abundance (1.56-2.65-fold) in the newly developed heterocysts. This is in accordance with previously published data [7, 12, 18] showing that in heterocysts, the down regulation of oxygenic photosynthesis leads to an increased demand of reducing power, which is produced from alternative pathways. The OPP pathway plays a particular important role in NADPH production during N_2_ fixation [59].

A reduction in the CO_2_ fixation machinery, including proteins involved in the Carbon Concentrating Mechanism (CCM) (CcmM, Npun_R3840; 0.48-0.09-fold) and all the enzymes in the Calvin-Benson-Bassham Cycle (0.26-0.02-fold; Additional file 1) was clearly evident in the heterocysts. This is in line with a previous suggestion that both structural components of the carboxysome and the subunits of RuBisCO are degraded in heterocysts during the differentiation process [60].

The lack of CO_2_ fixation in the heterocysts is compensated by import of reduced carbon, in the form of sucrose, from the neighboring vegetative cells [61]. Sucrose synthase (SusA, Npun_F1876), a bidirectional protein, which cleaves sucrose and provides sugar nucleotides for glycogen biosynthesis in the vegetative cells [62], was found at lower abundance in the heterocysts (0.15-fold). This is not surprising, as SusA is repressed by NtcA [63] and sucrose degradation in the heterocysts is instead, thought to be performed, by the unidirectional enzyme InvB [64]. Several other proteins involved in carbon storage/glycogen metabolism were also found at lower abundance (0.28-0.15-fold) in the newly formed heterocysts (e.g. GlgB, GlgC, GlgP, Npun_F0126 and Npun_F5814). The differential abundance of proteins involved in sucrose and glycogen metabolism support the suggestions from earlier studies [61, 62] that sucrose is transported into the newly formed heterocysts. Furthermore, it implies that this redirection of reduced sugars takes place at the expense of carbon storage, a common feature among N_2_-fixing cells [65, 66].

A large number of enzymes involved in both glycolysis and gluconeogenesis were also quantified (Additional file 1). Interestingly, three enzymes were each detected in two different isoforms. While the isoforms of Fructose-bisphosphate aldolases (Npun_F5584 and Npun_R0192) showed lower abundance in the heterocysts (0.16-0.13-fold), the isoforms of Fructose 1,6-bisphosphatase (Npun_F3917 Npun_F4023) and Glyceraldehyde-3-phosphate dehydrogenase (Npun_R0444 Npun_R0031) showed differential cell-type specific abundance. This reflects the different metabolic demands of heterocysts and vegetative cells and is likely due to the strong need for NADPH from the OPP pathway in the heterocysts. Two enzymes involved in acetyl-CoA synthesis, pyruvate dehydrogenase (Npun_R4179, Npun_F5580 and Npun_F3849) and acetyl-CoA synthetase (Npun_R6266) were also quantified. While all three subunits of pyruvate dehydrogenase showed no differential abundance, acetyl-CoA synthetase was found at lower abundance in heterocysts (0.12-fold), hence indicating that pyruvate dehydrogenase is the preferred pathway for acetyl-CoA synthesis in the heterocyst.

#### Cell-type specific regulation of proteins involved in stress, redox regulation and the unassigned metabolism

Given our interest in the redox regulated Cys-proteome, we also analyzed several proteins involved in redox regulation and detoxification of reactive oxygen species (ROS) in the data set (Additional file 1). The majority of these enzymes, including peroxidases, thioredoxin reductases, glutaredoxins and proteins involved in glutathione metabolism and glutathionlylation, were all found at lower abundance (0.65-0.08-fold) in the isolated heterocysts, as compared to the parental N_2_-fixing filaments. Significantly lower abundance levels of proteins, which are believed to be key players within redox regulation and oxidative stress responses in cyanobacteria, have also previously been discovered in the heterocyst proteomes of steady-state N_2_-fixing cultures of both *Anabaena* sp. PCC 7120 [18] and *N. punctiforme*[8]. We therefore suggest that different proteins control these processes within the heterocysts as compared to in vegetative cells. This is likely linked to cell-type specific differences in cellular and protein redox states and hence differences in redox regulation and ROS accumulation. Although ROS have been detected in both vegetative cells and heterocysts [67] results from our group and others indicate that the lowered oxygen tension and changes in photosynthetic and respiratory activities in the heterocysts would require different defensive strategies in heterocysts as compared to vegetative cells, see e.g. [68].

In cyanobacteria a number of flavodiiron proteins have shown to be of importance for different stress related conditions [69, 70]. We identified two of these proteins, Flv 1B (Npun_F4866) and Flv 3B (Npun_F4867), in much higher abundance (5.15-5.26-fold) in the newly formed heterocysts. This is in agreement with earlier reports from steady-state N_2_-fixing cultures of *N. punctiforme*[7] and *Anabaena* sp. PCC 7120 [18, 71]. These flavodiiron proteins are thought to protect oxygen sensitive enzymes in the heterocysts by reducing oxygen directly to water [71]. In contrast, two other isoforms of these flavodiiron proteins, Flv1 and Flv3, have been shown to protect the photosynthetic apparatus in vegetative cells against oxidative damage [71]. This is another example of a cell-type specific regulation of redox reactions that takes place in the two different types of cells within a diazotrophic filament.

In addition to the proteins discussed above, 15 proteins with an unknown function showed a higher abundance in the heterocysts as compared to their parental N_2_-fixing filaments (>2-fold; Table 3). Among these several have also been shown to be up-regulated (>2-fold) at the transcript level during the same developmental stage (24 hours after nitrogen depletion) in whole N_2_-fixing filaments [12]. Further research is currently ongoing to understand the roles of these proteins in relation to heterocyst physiology and N_2_ fixation.

**Table 3:** **Proteins of unknown function with a higher cell-type specific relative abundance in the newly** formed heterocysts of ***N. punctiforme*** as compared to N_2_-fixing filaments

RefSeq locus tag	Log _2_ ratio (Het/Fil)	Variability [%]
Npun_R0154	3.28	0.02
Npun_F5956	2.49	9.15
Npun_F1129	2.33	9.56
Npun_F1721	2.29	4.50
Npun_F0785	2.23	6.19
Npun_R0375	1.95	9.79
Npun_R1144	1.77	0.00
Npun_F4773	1.69	1.62
Npun_R4658	1.65	15.6
Npun_F1034	1.65	19.0
Npun_R0499	1.49	7.38
Npun_R3559	1.25	20.0
Npun_R4268	1.06	9.11
Npun_F4335	1.02	17.6
Npun_F0815	1.01	20.7

A summary of proteins with unknown function with higher relative abundance (>2-fold, i.e. Log_2_ >1) in heterocysts compared to their parental N_2_-fixing filaments (Additional file 1). The cell-type specificity indicates a specific role in the physiology of heterocysts.

In summary, we have here presented a quantitative cell-type specific proteomic data set from N_2_-fixing filaments of *N. punctiforme*. The data set contains a large subset of novel quantitative proteomic data, which have not been described previously. The transcriptional patterns of the pathways or the corresponding genes of individual proteins, presented here, have in several cases been described earlier, but mainly at the filament level. Hence, we were here able to verify the cell-type specificity of processes seen up-regulated at the filament level in N_2_-fixing cultures as compared to cultures cultivated with combined nitrogen.

### Comparative analysis of the differential cell-type specific proteomes in young and steady-state diazotrophic cultures

Comparing the presented data (Additional file 1) with cell-type specific proteomics data from steady-state diazotrophic cultures [7] gave us an insight into how the division of labor changes within the N_2_-fixing filaments over time. Our rational was to follow the change from a newly developed N_2_-fixing culture, with a synchronized population of functional heterocysts, to a steady-state culture, with a mixed heterocyst population of new and more mature heterocysts. As mentioned before, over 850 genes are differentially regulated in young as compared to steady-state N_2_-fixing cultures of *N. punctiforme*[12, 27]. This indicates a continuation of the transition into a steady-state culture also after the formation of functional heterocysts. Overall, the lower abundance of proteins involved in the core metabolism in newly formed heterocysts, compared to N_2_-fixing filaments, is to a large degree adjusted to even levels in heterocysts isolated from steady-state cultures (Table 2). The data comparison revealed a wide coverage of quantified core metabolic processes and enzyme families in both datasets. As mentioned above, a higher coverage of proteins involved in heterocyst specific metabolism is evident in the present study of newly formed heterocyst, while a lower coverage was detected of proteins involved in photosynthesis and ATP synthesis. Whether these differences reflect changes in the metabolism of the cells, the synchronicity of the heterocyst population or differences in the experimental methodologies remains to be determined. However, the use of ICAT and isolation of age-synchronized heterocysts clearly increased the accuracy and range of the quantified protein ratios and hence enabled a finer resolution of the cell-type specific differences within a diazotrophic filament.

Many of the 511 relatively quantified proteins (Additional file 1) were not quantified in the previous cell-type specific proteomics of steady-state diazotrophic cultures [7]. However, the 180 proteins that overlap between the two data sets facilitated a more in-depth comparative analysis (Additional file 4). As with the functional categories (Table 2), we detected a larger degree of proteins with a lower abundance in the newly developed heterocyst fraction compared to in the steady-state heterocyst fraction [7]. This could reflect a lowered heterocyst metabolism 24 h after combined nitrogen removal, prior to optimization of heterocyst metabolism towards a steady-state N_2_-fixating culture. However, a large number of these 180 proteins showed lower abundance in the heterocysts as compared to in their parental N_2_-fixing filaments in both data sets. Taken together a large number of the proteins identified in the two compared datasets showed a cell-type specific abundance that differed between the two developmental stages. This indicates that changes within the heterocyst proteome continue 24 hours after combined nitrogen depletion and is an important factor to complete the metabolic balance in a steady-state diazotrophic culture.

The proteins found at higher abundance in heterocysts of both young and steady-state cultures were to a large extent involved in N_2_ fixation, heterocyst development, respiration, energy production, PSI and the OPP pathway. In addition, several proteins of unknown function (e.g. Npun_F5956 and Npun_F1721) were also found at higher abundance in the heterocysts at both developmental stages. The heterocyst enrichment of these proteins of unknown function in both heterocyst populations strongly implies that they are part of the heterocyst’s physiology. Further investigations are therefore currently ongoing to determine their specific roles.

The 57 proteins found at lower abundance in the heterocysts, at both developmental stages (Additional file 4), are to a large extent involved in pathways that compete for energy and reducing power with processes that are enhanced in heterocysts. These include proteins involved in alternative nitrogen metabolism, specific cell wall enzymes, amino acid metabolism, phycobilisomes, reductive pentose phosphate pathway and carbon storage. In addition, a putative iron transporter (Npun_F4749) and several proteins of unknown function (e.g. Npun_AR289; Npun_R4965; Npun_R5610; Npun_R4472 and Npun_F1662) were also found at a lower abundance in the heterocysts of both young and steady-state N_2_-fixing cultures.

Another 17 proteins showed a lower cell-type specific abundance in heterocysts from steady-state cultures compared to their newly formed counterparts (Additional file 4). These include the global nitrogen regulator NtcA. NtcA has previously been shown to be strongly induced upon nitrogen limitation and decrease shortly after, both in heterocyst-forming [12] and non-heterocystous cyanobacteria [72]. Several ribosomal subunits were also detected at lower abundance in the steady-state heterocysts, as compared to their newly formed counterparts. We therefore suggest that while a strong down-regulation of amino acid biosynthesis is present in heterocysts in general, translation is still more active in the young heterocysts to support an efficient expression of the heterocyst specific proteome.

**Figure 3:**
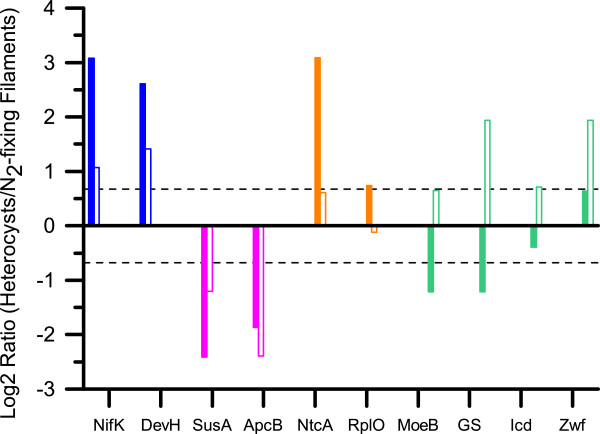
**Transitional changes in the cell-type specific relative abundance of proteins in young and steady-state N**_**2**_ **-fixing cultures.** The transitional changes in the relative cell-type specific abundance of a selected set of proteins quantified in both young (filled bars) and steady-state diazotrophic cultures (open bars) [7]. The color depicts the relative abundance of each protein based on their cell-type specificity in samples from young and steady-state cultures (blue – higher in heterocysts in both samples, pink – lower in heterocyst in both samples, orange – higher in newly formed heterocysts, green – higher in steady-state heterocysts). The dashed horizontal lines depict the Log_2_ +/-0.678 thresholds of 1.6-fold. Abbreviated protein names are used. For full name, see Additional file 1.

The remaining 67 proteins, including the anti-sigma factor antagonist Npun_R5561, were found with a higher relative abundance in heterocysts from steady-state N_2_-fixing cultures compared to newly formed heterocysts (Figure 3; Additional file 4). Two proteins involved in molybdopterin biosynthesis MoaB (Npun_R3942) and MoeB (Npun_F2020), which were found at lower abundance in newly formed heterocysts as compared to the parental N_2_-fixing filaments, were found at equal or moderately higher abundance in steady-state heterocysts (Figure 3). This might reflect a lowered synthesis rate of competing pathways involved in Mo-cofactor biosynthesis during the biosynthesis of the dinitrogenase specific FeMo-cofactor in the synchronized population of early heterocysts. It has previously been assumed that Mo is redirected towards the synthesis of the FeMo-cofactor during combined nitrogen deprivation; but to our knowledge this is the first evidence of a down regulation of enzymes in the competing molybdopterin pathway. A lower cell-type specific abundance of glutamine synthetase (GlnA/GS) was also evident in the newly formed heterocysts while it showed a higher abundance in heterocysts from steady-state diazotrophic cultures. Proteins involved in the TCA cycle (DhsB; GltA, Icd and AcoB) and the OPP pathway (e.g. zwf (Npun_F4025)), which were found at equal levels in newly formed heterocysts and their corresponding N_2_-fixing filaments, showed a higher cell-type specific abundance in steady-state heterocysts (Figure 3). This possibly reflects the need for a more diverse set of carbon skeletons in heterocysts of a steady-state N_2_-fixing culture, e.g. for nitrogen export and/or for the many processes temporarily down regulated during the early stages of heterocyst maturation.

Taken together, cell-type specific abundance of both single proteins and their corresponding metabolic pathways differed in heterocysts from young and steady-state N_2_-fixing cultures. This points to an incomplete metabolic shift in the newly formed heterocysts, related to both the central carbon and nitrogen metabolism. Indicating that the newly formed heterocysts, 24 h after nitrogen removal, are not yet fully adapted to its new N_2_-fixing metabolism.

### Newly formed diazotrophic cultures of *N. punctiforme*, correlation of quantitative proteomic and filament-level transcriptomic data

The corresponding transcripts of the protein encoding genes of 165 of the 511 proteins evaluated in this study were previously quantified by Christman *et al.*[12]. The transcriptomic data describes changes in N_2_-fixing filaments of *N. punctiforme*, 24 h after combined nitrogen depletion, as compared to ammonium-grown filaments. Of the overlapping data points 37% (61/165) showed a differential regulation at the transcript level, while 73% (121/165) were differentially regulated at the protein level (Additional file 5). Despite these differences, a high degree of correlation was detected between differentially regulated transcripts and proteins. Using the previously published cluster-analysis grouping of Christman *et al.*[12], we were able to corroborate the heterocyst specificity of many of the transcripts up-regulated in N_2_-fixing cultures, as compared to ammonium grown filaments. Transcripts up-regulated early following combined nitrogen deprivation (cluster 6) showed a mixed correlation between transcript levels and heterocyst specific protein levels, with 50% showing higher protein levels in the newly formed heterocysts. The strongest correlation, of transcripts and heterocyst specific protein levels, was detected in cluster 4, which included genes strongly transcribed after the establishment of a reduced oxygen atmosphere inside the developing heterocysts. Cluster 4 contains e.g. genes encoding the nitrogenase enzyme complex. In fact, all 21 transcripts, in cluster 4, correlated with heterocyst enriched protein levels. Our data clearly corroborate the hypothesis that the proteins encoded by these transcripts are involved specifically in the heterocyst metabolism. This also indicates a predominant transcriptional regulation of heterocyst related proteins expressed during this stage of heterocyst differentiation. Relatively few of the proteins detected with a lower abundance in the heterocysts as compared to their parental N_2_-fixing filaments were detected in the transcript data set of Christman *et al.*[12]. This is likely due to the difficulty of detecting heterocyst-specific negative changes in the abundance of transcripts also present in vegetative cells in filament based data sets. Not surprising as the abundance of the heterocyst specific transcripts has been diluted 10-20-times when analyzed at the filament level.

The analysis of the cell-type specific proteomes of isolated newly formed heterocyst and N_2_-fixing filaments allowed the assessment of the cell-type specificity of the previously reported differential transcript levels [12]. While large-scale transcriptomics of a multicellular cyanobacterium at the filament level gives valuable information on the general response to changes in the environment, a more cell-type specific strategy is needed to determine the division of labor between different cell-types.

## Conclusions

Here we present the first cell-type specific Cys-proteome from a heterocyst-forming cyanobacterium, representing cell-type specific differences in the abundance of proteins from the potential reversible Cys-modified redox proteome. This is in contrast to previous Cys-proteomic studies of cyanobacteria, which have mainly focused on the characterization of thioredoxin targets in unicellular cyanobacteria. The high coverage and differential regulation of proteins involved in several important biosynthetic pathways, within the core and heterocyst specific metabolism, indicate a wide distribution of redox-regulated thiols within the central metabolism of both vegetative cells and heterocysts of *N. punctiforme*. Furthermore, the data shows the strength of the ICAT approach to increase the knowledge of redox-regulated processes of cell-type specific importance.

The use of ICAT and the isolation of newly formed heterocysts at a synchronized developmental stage enabled good quantitative estimates of the differential abundance of processes at the cell-type specific level. It also enabled a better coverage of proteins involved in heterocyst differentiation than what has been reported from previous studies [7, 18]. In addition, the data presents a large set of proteins not covered in earlier cell-type specific data sets [7, 18], thereby increasing our understanding of the general heterocyst metabolism. These include novel proteins of putative importance, with both known and unassigned functions, which will require further investigation.

The cell-type specific metabolism in young and steady-state diazotrophic cultures show clear physiological differences, which extends beyond the induction of N_2_ fixation. This is evident by differentially regulated biochemical pathways and individual proteins in newly formed functional heterocysts, compared to their steady-state counterparts.

## Electronic supplementary material

Additional file 1: **Quantified cell-type specific relative abundance of proteins from isolated heterocysts as compared to their parental N**_**2**_ **-fixing filaments of** ***N. punctiforme*** **, 24 hours after removal of combined nitrogen.** The proteins are grouped based on manual curated functional groups [27]. Values above or below the Log_2_ +/-0.678 thresholds indicative of 1.6-fold are highlighted in color. (XLSX 214 KB)

Additional file 2: **Novel quantitative data from N**_**2**_ **-fixing cultures of** ***N. punctiforme*** **.** Protein cell-type specific relative abundance data from the present study, not previously represented in neither cell-type- or filament-based proteomic, nor transcriptomic, analyses of *N. punctiforme*[7, 12, 27]. The proteins are grouped based on manual curated functional groups [27]. Values above or below the Log_2_ +/-0.678 thresholds of 1.6-fold are highlighted in color. (XLSX 85 KB)

Additional file 3: **The comparative distribution of the cell-type specific protein quantitative ratios.** The relative abundance and distribution of cell-type specific ratios (Log_2_) of all quantified proteins (Heterocysts/N_2_-fixing filaments) in cultures 24 hours after removal of combined nitrogen (present study) and from steady-state N_2_-fixing filaments [7]. (PPTX 300 KB)

Additional file 4: **Comparative analysis of cell-type specific relative protein abundance levels in newly formed and steady-state diazotrophic cultures of** ***N. punctiforme*** **.** Proteins quantified from isolated heterocysts as compared to N_2_-fixing filaments of *N. punctiforme* from steady-state [7] and newly formed diazotrophic cultures (present study). The comparative analysis is based on the Log_2_ +/-0.678 thresholds of 1.6-fold used in both studies. (XLSX 37 KB)

Additional file 5: **Comparative analysis of cell-type specific relative protein abundance levels and filament transcript abundance levels in** ***N. punctiforme*** **in response to combined nitrogen limitation** ***.*** Transcript data from N_2_-fixing and ammonium-grown filaments of *N. punctiforme*, 24 hours after removal of combined nitrogen, were taken from Christman *et al.*[12]. This includes the transcript cluster grouping, which was based on the transcripts expression pattern over the first 24 hours following nitrogen step down [12]. The data was compared to cell-type specific protein abundance ratios of proteins detected in the present study. Values above or below the Log_2_ +/-0.678 significance thresholds of 1.6-fold are highlighted in color. (XLSX 33 KB)

## Abbreviations

ACN: Acetonitrile
COG: Clusters of orthologous group
Cys: Cysteine
ICAT: Isotope coded affinity tags
DAB: Denaturing alkylation buffer
FA: Formic acid
LC: Liquid chromatography
MS/MS: Tandem mass spectrometer
N. punctiforme: *Nostoc punctiforme* ATCC 29133-S
OPP pathway: Oxidative pentose phosphate pathway
PSI: Photosystem I
PSII: Photosystem II
ROS: Reactive oxygen species
RuBisCO: Ribulose-1,5-Bisphosphate Carboxylase/Oxygenase
SCX: Strong cation exchange
TCA cycle: The citric acid cycle
TCEP: Tris (2-carboxyethyl) phosphine.
