# Supplementary material for: Analysis of the early heterocyst Cys-proteome in the multicellular cyanobacterium Nostoc punctiforme reveals novel insights into the division of labor within diazotrophic filaments
Source: BMC Genomics. 2014 Dec 4;15(1):1064. doi: 10.1186/1471-2164-15-1064 (PMC4363197; doi:10.1186/1471-2164-15-1064)
Supplement: Supplementary file 3 — Additional file 3: The comparative distribution of the cell-type specific protein quantitative ratios. The relative abundance and distribution of cell-type specific ratios (Log2) of all quantified proteins (Heterocysts/N2-fixing filaments) in cultures 24 hours after removal of combined nitrogen (present study) and from steady-state N2-fixing filaments [7]. (PPTX 300 KB) [file 12864_2014_6948_MOESM3_ESM.pptx]

## Slide 1
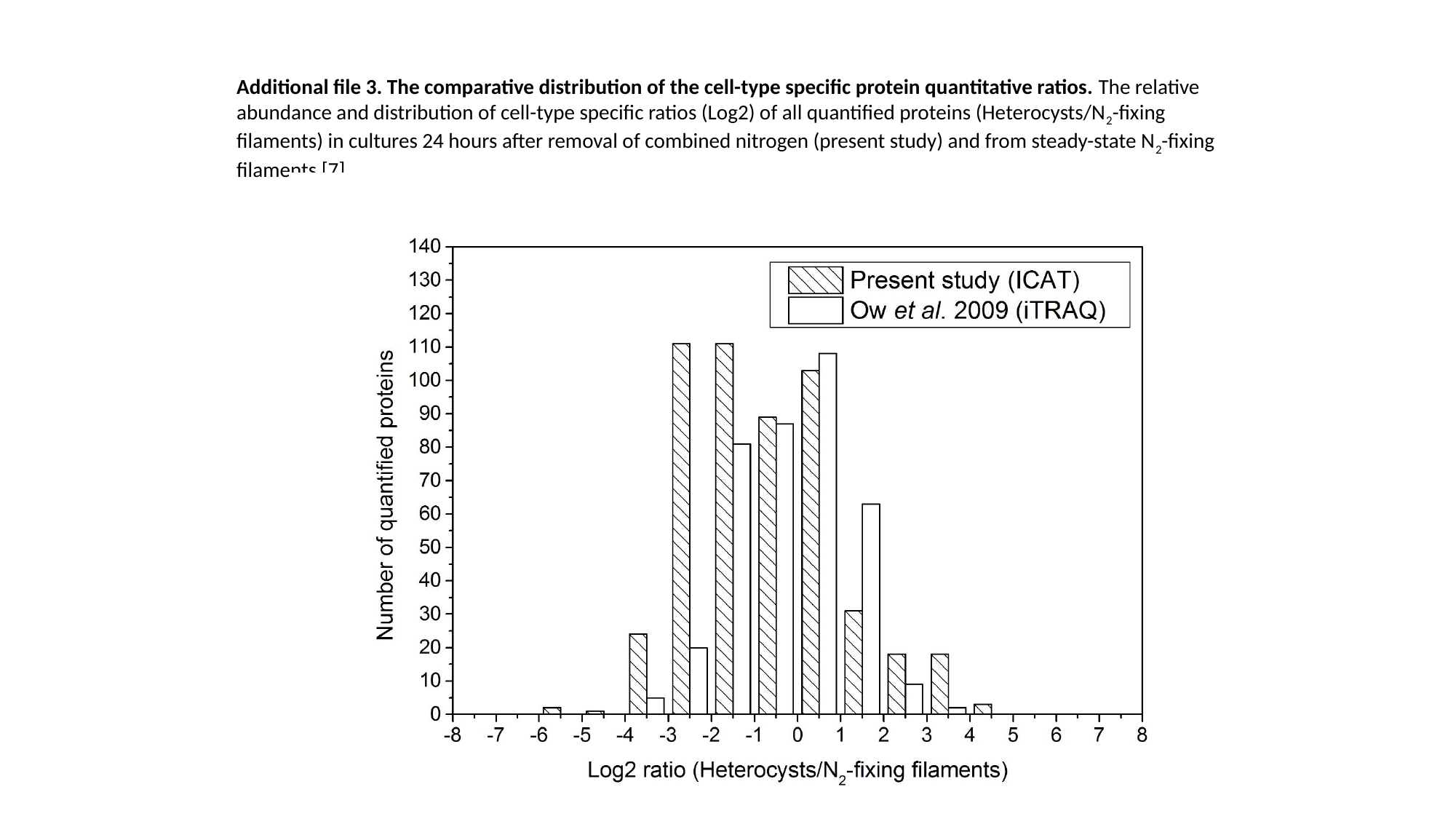

Additional file 3. The comparative distribution of the cell-type specific protein quantitative ratios. The relative abundance and distribution of cell-type specific ratios (Log2) of all quantified proteins (Heterocysts/N2-fixing filaments) in cultures 24 hours after removal of combined nitrogen (present study) and from steady-state N2-fixing filaments [7].
